# Psilocybin Dispensaries and Online Health Claims in Canada

**DOI:** 10.1001/jamanetworkopen.2025.2853

**Published:** 2025-04-01

**Authors:** Jenna Matsukubo, Sarah Dickson, Jennifer Xiao, Erik Loewen Friesen, Marco Solmi, Jess G. Fiedorowicz, Benedikt Fischer, Daniel T. Myran

**Affiliations:** 1 Faculty of Medicine, University of Ottawa, Ottawa, Ontario, Canada; 2 Faculty of Health Sciences, Queen’s University, Kingston, Ontario, Canada; 3 Ottawa Hospital Research Institute, Ottawa, Ontario, Canada; 4 Institut du Savoir Montfort, Ottawa, Ontario, Canada; 5 Temerity Faculty of Medicine, University of Toronto, Toronto, Ontario, Canada; 6 SCIENCES Lab, Department of Psychiatry, University of Ottawa, Ottawa, Ontario, Canada; 7 Department of Mental Health, The Ottawa Hospital, Ottawa, Ontario, Canada; 8 Department of Child and Adolescent Psychiatry, Charité Universitätsmedizin, Berlin, Germany; 9 School of Epidemiology and Public Health, Faculty of Medicine, University of Ottawa, Ottawa, Ontario, Canada; 10 Department of Psychiatry, University of Toronto, Toronto, Ontario, Canada; 11 Faculty of Health Sciences, Simon Fraser University, Vancouver, British Columbia, Canada; 12 Research & Graduate Studies, University of the Fraser Valley, Abbotsford, British Columbia, Canada; 13 Waypoint Research Institute, Waypoint Centre for Mental Health, Penetanguishene, Ottawa, Canada; 14 Department of Family Medicine, University of Ottawa, Ottawa, Ontario, Canada; 15 Bruyère Health Research Institute, Ottawa, Ontario, Canada

## Abstract

**Importance:**

There is growing societal interest in and use of psilocybin. While psilocybin in Canada is illegal outside of clinical trials, there have been anecdotal reports of increasing access via unregulated online purchases and retail dispensaries.

**Objective:**

To describe access to and the characteristics of psilocybin dispensaries across Canada and the health claims and warnings made on dispensary websites.

**Design, Setting, and Participants:**

This cross-sectional study used systematic web searches and media reports to identify psilocybin dispensaries operating in Canada in May 2024. Data analysis was performed from June 17 to August 29, 2024.

**Main Outcomes and Measures:**

Descriptive and geospatial analyses were used to identify the psilocybin dispensary characteristics, product types, and store distribution. Content analysis assessed the nature and frequency of health claims and warnings on websites.

**Results:**

As of May 2024, 57 psilocybin dispensaries were identified in Canada (0.18 dispensaries per 100 000 individuals aged ≥15 years) in 15 of Canada’s 42 major urban cities (35.7%). Approximately 815 628 (2.6%) of Canadians lived within 1 km of a dispensary. Only 4 of 13 provinces and territories had a dispensary, with most in Ontario and British Columbia. Of the 57 stores, 35 (61.4%) were part of a chain (≥2 stores owned by a single company) and 52 (91.2%) had an online presence. Stores sold a wide variety of products, including dried mushrooms (100.0%), microdosing capsules (97.8%), psilocybin-infused chocolate (91.3%) and gummies (93.4%), and most stores (65.2%) sold products mimicking popular food brands. Among stores with websites, 86.4% claimed mental health benefits of psilocybin (eg, alleviating anxiety). While 86.4% of websites provided health warnings, relevant warnings, such as those about use while driving (9.1%), during pregnancy (13.6%), or in individuals with a history of psychosis, schizophrenia, or bipolar disorder (31.8%) were rare.

**Conclusions and Relevance:**

In this study, psilocybin retailers, who were present in over a third of major Canadian cities, made a variety of unverified health claims and lacked warnings of potential harms, suggesting the need for greater regulatory measures to protect the public.

## Key Points

**Question:**

What level of access is there to unregulated psilocybin dispensaries in Canada and what kind of health claims and warnings are made by dispensaries?

**Findings:**

In this cross-sectional study of all psilocybin dispensaries operating in Canada, we identified 57 dispensaries and 15 of Canada’s 42 major urban cities contained 1 or more psilocybin dispensaries. Most dispensaries had a website that offered online sales while making unsubstantiated health claims and important omissions of risk.

**Meaning:**

These results suggest that further regulatory action may be warranted in response to growing access to unlicensed psilocybin dispensaries and product promotion.

## Teaser

This cross-sectional study examines the presence of psilocybin dispensaries in Canada, the kinds of products available, and the health claims and warnings that appear on their websites.

## Introduction

Over the past 2 decades, there has been a revival in public and scientific interest in the potential therapeutic use of psychedelics for the treatment of mental and substance use disorders.^1,2^ Psychedelics, often referred to as classic or serotonergic hallucinogens, include lysergic acid diethylamide, n,n-dimethyltryptamine, and psilocybin, which may colloquially be called magic mushrooms or shrooms. ^3^ Evidence from randomized clinical trials suggests that psychedelic-assisted therapy may have utility for treatment-resistant depression, posttraumatic stress disorder (PTSD), and alcohol use disorder.^4,5,6,7,8,9^ These findings have led to growing medical and societal interest and corresponding use of psychedelics.^10,11^ In North America, hallucinogen use in adults has been increasing since the mid-2010s,^12,13,14,15^ and between 2018 and 2021, the percentage of individuals aged 19 to 30 years who reported past-year non–lysergic acid diethylamide hallucinogen use (eg, psilocybin) nearly doubled in the US from 3.4% to 6.6%.^16^ As interest and use of hallucinogens have increased, there have been anecdotal reports on the proliferation of psilocybin dispensaries and online retail websites in recent years in North America.^17,18,19,20,21^ There is limited information about access to these dispensaries and the medical information being presented to the public through sale.

Hallucinogens remain illegal in most of North America, although the regulatory picture is rapidly changing.^22,23,24,25^ In the US, Oregon and Colorado became the first states to create legislative frameworks for psilocybin service centers where clients can legally buy and consume psilocybin under supervision.^26,27,28^ In Canada, psilocybin products remain unlicensed. However, there are 2 primary pathways for individuals to obtain authorization from Health Canada to access psilocybin legally: through participation in clinical trials and the Special Access Program.^24^ The Special Access Program allows health care practitioners to request access to nonmarketed drugs that have shown therapeutic potential in clinical trials or have been approved in other countries for serious or life-threatening conditions when conventional therapies are ineffective, unsuitable, or unavailable.^24^ Regulatory amendments made in January 2022 enabled health care practitioners to request access to restricted drugs, such as psilocybin, through the Special Access Program.^29^

While the production, sale, and possession of psilocybin remain unlicensed in Canada unless authorized by Health Canada, brick-and-mortar psychedelic dispensing stores and online sellers are widely reported across Canada.^17,18,19,20^ To our knowledge, no research has been conducted on the number and characteristics of these stores, including their location, their product offerings, website content, and online marketing. To address this gap, this study aimed to describe access to and the characteristics of psilocybin dispensaries in Canada and the health claims and health warnings made on dispensary websites.

## Methods

Our study used publicly available data without identifying information, and no ethics approval or informed consent is required in Canada. This study followed the Strengthening the Reporting of Observational Studies in Epidemiology (STROBE) reporting guideline.

### Data Collection and Sources

#### Psilocybin Stores

We identified the physical and online presence of psylocibin dispensaries across Canada at 2 time points, in November 2023 and May 2024, using a 2-step approach applying similar techniques to those previously used to map electronic cigarette and cannabis dispensaries.^30,31,32^ First, we completed online searches (Google and Google Maps) with the following terms and logic: the province or city name (eg, Ontario or Toronto); and magic mushroom, magic mushroom store, magic mushroom dispensary, and shroom delivery. The cities included were the 42 major urban population centers in Canada (census metropolitan areas) and we expanded search areas to account for surrounding and rural regions. Second, we searched for media reports (eg, a story about police finding an illegal store) on stores to find additional locations. All store locations and operating statuses were validated using a second source (including online business listings, local media reports, or social media sites, including posts on social media). In addition, online website searches facilitated snowball searches of additional physical store locations (eg, as part of a franchise).

To be included in the analysis, a store had to advertise the selling of psilocybin products at a physical location. We recorded the address and city in which the store was located noting whether a store link was available, whether it was a part of a chain, and the source of information (eg, web search, news sources). Chain status was determined based on dispensary name. If a store had a website, we recorded the following content: location, presence and method of age gate verification, availability of delivery, products sold, proposed health claims, and disclaimers on potential harms. After the initial extraction, the type of psilocybin sold (dried, microdose capsule, infused chocolate, infused gummy, or other) and resemblance to mass market brands were extracted using the same procedure. All data on the characteristics were dual extracted and verified (J.M. and J.X.). Language relating to health claims and potential harms was extracted from all sections of psilocybin websites, including product descriptions. Websites for psilocybin chains were only extracted once, and the results were applied to all chain locations.

### Statistical Analysis

#### Characteristics of Stores

We summarized psilocybin store characteristics, including operating status, chain status and type, online presence and type, delivery, age gating, types of products sold, and presence of health claims and warnings. This was reported for all stores and stratified by chain size.

#### Access to Psilocybin Stores

We obtained the annual estimated population aged 15 years or older on July 1, 2021, for each jurisdiction in Canada from Statistics Canada and applied the population to all months within the calendar year. We defined neighborhoods as dissemination areas (n = 56 589 in Canada), which are the smallest geographic unit for which census data are released in Canada.^33^ Psilocybin retail outlets were geocoded based on street address (ArcGIS World Geocoding Network; Esri). We examined the presence of psilocybin stores in the 42 major urban cities (census metropolitan areas) in Canada. Psilocybin stores per capita were reported using census metropolitan area populations of individuals aged 15 years or older from the 2021 census. For Toronto and Vancouver, per capita stores were reported for both the census subdivision and census metropolitan areas, the latter of which contain neighboring municipalities. The municipality in which a psilocybin store was located was determined by postal code.

To evaluate the population exposed to psilocybin stores, we calculated the number of neighborhoods and their local population as per the latest Canadian census completed in 2021 within 1 km of each psilocybin store. This was accomplished by creating 1-km buffer zones around each store (using the CanMap 2021 road network file and the Network Analyst Tool in ArcGIS Pro) and then counting the number of neighborhood centroids that fell within the buffer zone. For neighborhoods that were within 1 km of a psilocybin store, we also tabulated the total number of stores within 1 km of the neighborhood centroid to assess for neighborhoods that had access to multiple stores.^34^

#### Content Analysis of Websites

We used a content analysis method to identify common terms and themes present in psilocybin website content. Text was coded into themes (eg, health claim relating to bipolar disorder treatment) that were developed iteratively by the team. Health claims were broadly categorized into mental health–, substance use–, nonmedical or wellness–, and physical health–related claims. Health harm warnings were categorized into cautions for specific populations, potential risks and adverse effects, safety and use precautions, and quality assurance. We consulted with 2 psychiatrists (M.S. and J.G.F.) to determine the relevance of the themes to clinical practice and incorporated feedback to finalize themes and subthemes. The data were independently coded by 2 of us (J.M. and S.D.), and conflicts were resolved by another (D.T.M.) where necessary. After coding, salient quotations that were representative of each thematic category were selected by the research team. Data analysis was performed from June 17, 2024, to August 29, 2024.

## Results

### Description of Psilocybin Stores 

From November 2023 to May 2024, we identified 68 psilocybin stores operating in Canada. Between the time of the 2 extractions, 11 of the stores open in November 2023 had closed, leaving 57 in operation (Table 1). Of the 11 stores that closed, most (72.7% [8]) were independent stores or part of small chains. Of the stores that remained open, most (61.4% [35]) were part of a chain. We identified 4 small provincial chains that included a total of 9 stores, 1 large provincial chain comprising 6 stores, 1 small national chain comprising 2 stores, and 1 large national chain operating 18 (31.6%) stores. Among the 57 psilocybin stores, 52 (91.2%) had an online presence. Of the stores with an online presence, most only had a website (65.4%), 2 stores only had social media (3.8%), and the remaining stores had both (30.8%). Less than half (48.0%) of the stores with a website had an age verification step for site entrance. These age gates all appeared as separate pop-ups asking users to check the box if older than 19 years; none required identification or registration for age confirmation. Additionally, most of the stores with websites (n = 50) provided delivery services for their products (88.0%), made health claims (90.0%), and made health warnings (90.0%). Half of the stores with websites also sold substances other than psilocybin-containing products. Among the stores that sold other substances (n = 25), the most commonly co-sold products were the psychedelic n,n-dimethyltryptamine (92.0%) and cannabis (80.0%). In comparison with independent stores, large chain stores were more likely to have an online presence (100.0% vs 77.3%), require age verification to enter the website (75.0% vs 13.3%), provide online delivery services (100.0% vs 73.3%), make health claims and health warnings (100.0% vs 93.3%), and sell other substances besides psilocybin-containing products (75.0% vs 26.7%).

**Table 1.** Characteristics of Canadian Psilocybin Retail Stores

Characteristic	No. (%)			
	All stores	Independent^a^	Small chain^b^	Large chain^c^
Online presence				
No. with data	57	22	11	24
No	5 (8.8)	5 (22.7)	0	0
Yes	52 (91.2)	17 (77.3)	11 (100)	24 (100)
Online type				
No. with data	52	17	11	24
Website only	34 (65.4)	9 (52.9)	7 (63.6)	18 (75.0)
Social media only	2 (3.8)	2 (11.8)	0	0
Website and social media	16 (30.8)	6 (35.3)	4 (36.4)	6 (25.0)
Age gate				
No. with data	50	15	11	24
No	26 (52.0)	13 (86.7)	7 (63.6)	6 (25.0)
Yes	24 (48.0)	2 (13.3)	4 (36.4)	18 (75.0)
Online delivery				
No. with data	50	15	11	24
No	6 (12.0)	4 (26.7)	2 (18.2)	0
Yes	44 (88.0)	11 (73.3)	9 (81.8)	24 (100)
Health claims				
No. with data	50	15	11	24
No	5 (10.0)	1 (6.7)	4 (36.4)	0
Yes	45 (90.0)	14 (93.3)	7 (63.6)	24 (100)
Health warning				
No. with data	50	15	11	24
No	5 (10.0)	1 (6.7)	4 (36.4)	0
Yes	45 (90.0)	14 (93.3)	7 (63.6)	24 (100)
Type of psilocybin product sold				
No. with data	46	13	9	24
Dried mushroom	46 (100)	13 (100.0)	9 (100)	24 (100)
Microdosing capsule	45 (97.8)	12 (92.3)	9 (100)	24 (100)
Psilocybin-infused chocolate	42 (91.3)	9 (69.2)	9 (100)	24 (100)
Psilocybin-infused gummy	43 (93.4)	10 (76.9)	9 (100)	24 (100)
Psilocybin-infused tea	26 (56.5)	6 (46.2)	2 (22.2)	18 (75.0)
Other psilocybin infused edible	16 (34.8)	6 (46.2)	2 (22.2)	24 (100)
Branded psilocybin copycat edible	30 (65.2)	4 (30.8)	2 (22.2)	18 (75.0)
Co-sale of other products				
No. with data	50	15	11	24
No	25 (50.0)	11 (73.3)	8 (72.7)	6 (25.0)
Yes	25 (50.0)	4 (26.7)	3 (27.3)	18 (75.0)
Co-sale products^d^				
No. with data	25	4	3	18
Cannabis	20 (80.0)	2 (50.0)	0	18 (100)
DMT	23 (92.0)	2 (50.0)	3 (100)	18 (100)
LSD	3 (12.0)	0	3 (100)	0
Coca leaf	3 (12.0)	0	3 (100)	0

Abbreviations: DMT, n,n-dimethyltryptamine; LSD, lysergic acid diethylamide.

^a^ Independent: stores that are not part of a chain.

^b^ Small chain: stores that are part of a chain with fewer than 5 stores.

^c^ Large chain: stores that are part of a chain with 5 or more stores.

^d^ The percentages for co-sale products exceed 100% because some stores sold multiple products.

### Psilocybin Products Sold

We collected data on the type of psilocybin products sold from 46 of the 50 stores with a website (2 stores closed before product extraction, 2 stores did not specify the type of products sold). Overall, stores sold a wide range of products, including dried mushrooms (100.0%), microdosing capsules (97.8%), psilocybin-infused chocolate (91.3%), psilocybin-infused gummies (93.4%), infused dried tea (56.5%), or other infused products (34.8%), such as premixed psilocybin or infused hot cocoa powder. Most stores (65.2%) offered 1 or more psilocybin-infused product specifically designed to mimic a common brand-name snack food item (eg, Mushtella, a Nutella spread mimic; a psilocybin-infused tea mimicking the Arizona Iced Tea brand; and psilocybin-infused chocolate bars mimicking brands such as Skor or Reese’s Peanut Butter Cup).

### Access to Psilocybin Retail Stores

Table 2 presents the absolute and number of stores per capita (individuals aged ≥15 years) by province and census metropolitan area. As of May 2024, there were 0.18 psilocybin retail stores open in Canda per 100 000 individuals aged 15 years or older (n = 57 stores) with 35.7% of the stores located in major urban centers (15 of 42). Across the country, 815 628 individuals (2.6%) in the population were within 1 km of a psilocybin dispensary. Of the 13 Canadian jurisdictions (provinces and territories), psilocybin dispensaries were located within only 4 provinces (Ontario, Quebec, British Columbia, and Manitoba), with the first 3 provinces listed being the largest in Canada by population. Most psilocybin stores (96.5%) were located in Ontario (n = 38 [0.32 stores per 100 000 individuals aged ≥15 years]) and British Columbia (n = 17 [0.4 stores per 100 000 individuals aged ≥15 years]), while Quebec (n = 1 [0.01 stores per 100 000 individuals aged ≥15 years]) and Manitoba (n = 1 [0.09 stores per 100 000 individuals aged ≥15 years]) only had 1 store in each. There was wide variation between municipalities in the percentage of the population aged 15 years or older that lived within 1 km of a psilocybin dispensary. In Montreal, 0.02% of the census metropolitan area population lived within 1 km of a psilocybin dispensary. In comparison, 15.8% of the Toronto census subdivisions and 37.9% of the Vancouver census subdivisions of the population aged 15 years or older lived within 1 km of a psilocybin dispensary. Stores also displayed clustering in Toronto, with some neighborhoods having up to 4 stores within 1 km.

**Table 2.** Psilocybin Stores Per Capita and Distribution Across Canadian Dissemination Areas

Province or census metropolitan area	Population aged ≥15 y (2021)	No. of stores open May 2024	Stores per 100 000	Number of DAs (2021)^a^	DAs within 1 km of a store (% total)	Population within 1 km of a store (% total)	Maximum stores within 1 km of DAs with 1 store
Canada	30 979 186	57	0.18	57 932	1256 (2.17)	815628 (2.63)	4
Ontario	11 972 147	38	0.32	20416	844 (4.13)	555021 (4.64)	4
Toronto (CMA)^b^	5 237 175	18	0.34	7711	539 (6.99)	395283 (7.55)	4
Toronto (CSD)^c^	2 410 061	17	0.71	3739	515 (13.77)	377720 (15.67)	4
Hamilton	659 369	4	0.61	1211	88 (7.27)	37799 (5.73)	2
Ottawa-Gatineau	947 969	6	0.63	2045	114 (5.57)	67574 (7.13)	1
Barrie	176 656	2	1.13	333	20 (6.01)	9957 (5.64)	2
Kitchener-Cambridge-Waterloo	477 257	2	0.42	747	7 (0.94)	3525 (0.01)	1
London	453 836	1	0.22	680	7 (1.03)	4158 (0.92)	1
St Catharines-Niagara	370 804	2	0.54	245	12 (4.9)	5437 (1.47)	1
Brantford	119 487	1	0.84	597	12 (2.01)	6137 (5.14)	1
Oshawa	338 461	1	0.3	759	8 (1.05)	5903 (1.74)	1
Windsor	353 835	1	0.28	663	16 (2.41)	7801 (2.2)	1
Quebec	7 110 473	1	0.01	13731	2 (0.01)	621 (0.02)	1
Montreal	3 570 007	1	0.03	6538	2 (0.03)	621 (0.02)	1
Manitoba	1 089 218	1	0.09	2223	10 (0.45)	6866 (0.63)	1
Winnipeg	693 558	1	0.14	1243	10 (0.8)	6866 (0.99)	1
British Columbia	4 283 979	17	0.4	7809	400 (5.12)	253741 (5.92)	2
Vancouver (CMA)^b^	2 270 775	15	0.66	3585	384 (10.71)	245637 (10.82)	2
Vancouver (CSD)^c^	591 678	12	2.03	1013	348 (34.35)	224149 (37.88)	2
Kamloops	97 172	1	1.03	171	11 (6.43)	5213 (5.36)	1
Nanaimo	99 064	1	1.01	179	5 (2.79)	2891 (2.92)	1

Abbreviations: CMA, Census Metropolitan Area; CSD, Census Subdivision (municipalities); DA, dissemination area.

^a^ DA is a small, relatively stable geographic unit composed of 1 or more adjacent dissemination blocks with an average population of 400 to 700 persons based on data from the previous Census of Population Program.

^b^ A CMA is a geographic region consisting of 1 or more adjacent municipalities centered around a population core of at least 50 000 people, with a total population of 100 000 or more.

^c^ A CSD is the general term for municipalities (as determined by provincial/territorial legislation) or areas treated as municipal equivalents for statistical purposes.

### Health Claims and Health Warnings

Table 3 reports the percentage of types of health statements, and a representative statement, across websites from psilocybin dispensaries, where the denominator is the number of unique websites (n = 22). The eTable in Supplement 1 lists additional representative quotes from the websites.

**Table 3.** Types of Health Statements Featured on Psilocybin Websites

Health claims or health warnings	Websites making health statements, No. (%)^a^	Quotations^b^
Mental health claims	19 (86.4)	NA
Medical claims associated with psychedelic-assisted therapy	5 (22.7)	Individuals who suffer from debilitating conditions such as depression and anxiety have been shown to get better in as little as 1 month by taking prescribed psychedelic mushroom dosages. However, you should note that the benefits are more profound and noticeable if the dosage is incorporated with other therapeutic sessions with trained professionals.
Anxiety and anxiety disorder	18 (81.8)	Some people with social anxiety or generalized anxiety disorder have reported a reduction in their symptoms after microdosing.
Depression, MDD, TRD	17 (77.3)	One of the most promising areas of my research has been in the realm of depression. Our studies have consistently shown that even a single dose of psilocybin can lead to rapid and enduring antidepressant effects. Participants in our trials have reported significant alleviations in their depressive symptoms, with many experiencing relief that lasts months post treatment.
PTSD	16 (72.7)	Psilocybin is a potent tool to cure severe cases of depression and PTSD.
End-of-life distress and anxiety associated with terminal illness	4 (18.2)	Patients, many of whom grapple with the profound anxiety of their mortality, have reported significant reductions in their distress levels after psilocybin-assisted therapy sessions.
SUD claims	10 (45.5)	NA
SUD (general)	10 (4.5)	For some, microdosing helps people quit habits, such as smoking cigarettes, stop using other drugs or drinking alcohol.
AUD	7 (31.8)	Some users have reported reduced cravings for addictive substances, such as alcohol or nicotine, after microdosing with magic mushrooms.
Nonmedical claims	19 (86.4)	
Increased focus and productivity	17 (77.3)	Microdosing may also help with temporary focus, allowing a person to work on a big project without their mind wandering.
Increased creativity and open-mindedness	18 (81.8)	They can boost your creativity, sharpen your focus, and help you concentrate deeply. Flow state achievement: they are perfect for creative people and professionals who want to perform better.
Spiritual experience, self-reflection, personal growth, and ego death	16 (72.7)	Many people use magic mushrooms for their hallucinogenic properties, seeking altered states of consciousness, introspection, and enhanced sensory perception. These experiences can be profound and have been described as spiritually enlightening or therapeutic.
Wellness flourishing	18 (81.8)	[This product is] known for feelings of euphoria and happiness. Feelings of love, acceptance, interdependence, and the oneness of all things.
Psychedelic effects	17 (77.3)	Experience a world where time and space take on new meanings, offering insights into the depths of your psyche.
Physical health claims	10 (45.5)	NA
Pain relief	8 (36.4)	Microdosing benefits: relief from menstrual pain.
Headaches, migraines	7 (31.8)	Microdosing benefits: reduction in cluster headaches, reduction in migraine headaches.
Anti-inflammation and tissue regeneration	3 (13.6)	Research suggests psilocybin could even help repair brain damage from trauma or disease. It’s fascinating to think how these natural substances might aid in mind healing.
Other (eating disorder, insomnia, sleep disorder, OCD, ADHD)	15 (68.2)	Microdosing benefits: boosted energy, addiction treatment, decreased anxiety, depression and PTSD relief, eating disorder treatment.
Evidence for health claims	2 (9.1)	NA
Referenced an academic study	2 (9.1)	Goodwin et al,^9^ 2022
Presence of health warnings	16 (86.4)	NA
Cautions about use in specific populations	12 (54.5)	NA
Children	7 (31.8)	Keep out of the reach of children and pets!
Pregnancy/breastfeeding	3 (13.6)	Consult a health care practitioner prior to use if you are breastfeeding.
History of psychosis, bipolar disorder, schizophrenia	7 (31.8)	People with a history of psychotic disorders, such as schizophrenia, bipolar or other disorders, may also want to avoid microdosing, as the practice may be too stimulating.
History of mental health conditions	11 (50.0)	Exercise caution if you have a history of mental health conditions. Magic mushrooms may have adverse effects in such cases.
Potential adverse effects	17 (77.3)	NA
Triggering psychosis, latent schizophrenia, and paranoia	9 (40.9)	Psilocybin can sometimes trigger psychological distress, such as anxiety, panic attacks, or psychosis. This is more likely to happen in people who have a history of mental illness.
Triggering latent bipolar disorder	1 (4.5)	Risk of triggering latent mental health issues such as schizophrenia or bipolar disorder in susceptible users.
Bad trip (increased anxiety, panic attacks, fear)	13 (59.1)	Doses over 3000 mg will produce intense psychedelic effects with a higher potential for feelings of fear and anxiety, usually without increased therapeutic benefit.
GI symptoms (nausea, vomiting, cramps)	9 (40.9)	If you experience nausea: consult a health care professional, start with a lower dose, and create a comfortable setting for a positive experience.
Increased HR and BP	4 (18.2)	Magic mushrooms contain psychoactive compounds that alter perception. However, these changes come with physical risks...there’s an increase in heart rate and blood pressure. For those with heart conditions, this poses a significant risk.
Nightmares, insomnia	7 (31.8)	Using a microdose in the evening can disrupt sleep patterns in some people.
Disruption in mood	8 (36.4)	Usually, mushrooms containing psilocybin change mood and sense and are sometimes not enjoyable. User emotions can be intense and terrifying.
Organ failure, death	1 (4.5)	One of the most severe risks associated with using any type of mushroom is poisoning—mistaking toxic varieties for safe ones. Symptoms range from mild gastrointestinal distress to severe organ failure and even death in extreme cases.
Hallucinations and altered perception	17 (77.3)	This compound is responsible for the hallucinations experienced during a mushroom trip because it affects the prefrontal cortex part of the brain where cognition occurs.
Potential to cause dependence	1 (4.5)	Magic mushrooms are not considered physically addictive, but they can be habit-forming in some cases. They should be used with care and respect.
Safety and usage precautions	14 (64.0)	NA
Do not mix with alcohol, cannabis, or other substances	6 (27.3)	Never mix psilocybin mushrooms with alcohol, weed, or other substances. It can have unpredictable and unpleasant effects.
Interactions with antidepressants and mood stabilizers	5 (22.7)	Do not combine with alcohol, SSRIs or SNRIs.
Do not drive, swim, climb, or engage in any risky behavior	2 (9.1)	Do not drive or operate heavy machinery.
Consult a health care professional	6 (27.3)	If you take any medications, especially antidepressants or mood stabilizers, consult your doctor before using magic mushrooms.
Start with a low dose	12 (54.5)	If you are new to shrooms, start with a low dose and eat something before trying them. In this way, you will check how shrooms work on you and avoid unpleasant feelings in the guts.
Have a trip sitter with you	6 (27.3)	It is recommended to have a trusted friend or professional by your side as a trip sitter.
Consider using in the presence of a therapist	5 (22.7)	Have a sober friend near you, or even invite a professional therapist to get as many insights from your trip as possible.
Product safety and quality assurance	9 (40.9)	NA
Quality control assurance	9 (40.9)	Our magic mushrooms are safe to consume. We prioritize safety, sourcing from trusted cultivators using organic practices. Our products undergo rigorous testing for purity and potency.

Abbreviations: ADHD, attention-deficit/hyperactivity disorder; AUD, alcohol use disorder; BP, blood pressure; GI, gastrointestinal; HR, heart rate; MDD, major depressive disorder; NA, not applicable; OCD, obsessive-compulsive disorder; PTSD, posttraumatic stress disorder; SNRIs, serotonin-norepinephrine reuptake inhibitors; SSRIs, selective serotonin reuptake inhibitors; SUD, substance use disorder; TRD, treatment-resistant depression.

^a^ The statements are presented verbatim from the psilocybin store websites.

^b^ The number of websites with presence of health claims was 19 (86.4%).

### Health Benefit Claims

**Figure 1.**
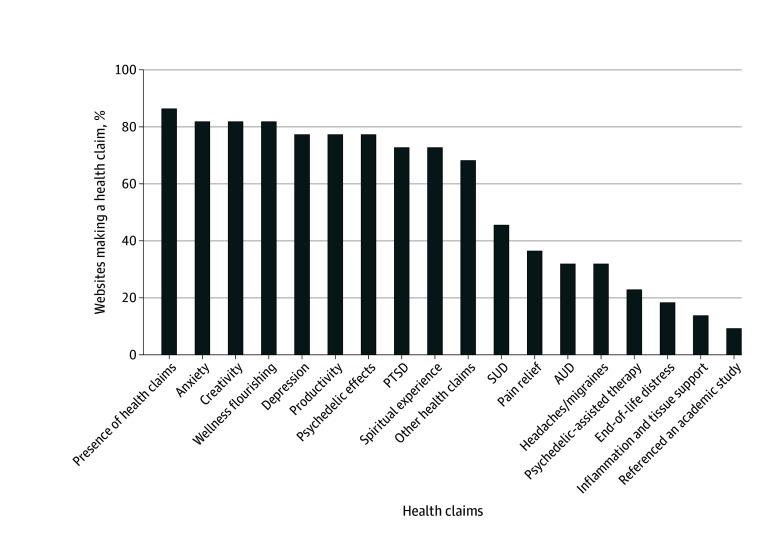
Types of Health Claims Featured on Psilocybin Websites AUD indicates alcohol use disorder; PTSD, posttraumatic stress disorder; and SUD, substance use disorder

The proportions of websites making specific health claims are outlined in Figure 1. Overall, 86.4% of websites made at least 1 mental health claim. The most common of these were about alleviating symptoms of anxiety (81.8%), depression (77.3%), and PTSD (72.7%). Health claims for treatment of substance use issues were less common (45.0%). Other mental health conditions mentioned included end-of-life distress (18.2%), obsessive-compulsive disorder (18.2%), attention-deficit/hyperactivity disorder (18.2%), and eating disorders (4.5%). Only 22.7% of websites specifically mentioned mental health benefits associated with psilocybin in the context of psychedelic-assisted therapy.

Most (86.4%) websites also made nonmedical claims, with increased creativity (82%) and wellness improvement (81.8%) being the most common, followed by increased productivity (77.3%), psychedelic effects (77.3%), and spiritual experience (72.7%). Claims related to physical health were less common than mental health and nonmedical claims; nevertheless, websites made claims about pain relief (36.4%), headache and migraine relief (31.8%), and anti-inflammation and tissue regeneration (13.6%). Overall, only 2 websites (9.1%) referenced academic studies as supportive evidence for a health claim.

### Health Warnings

**Figure 2.**
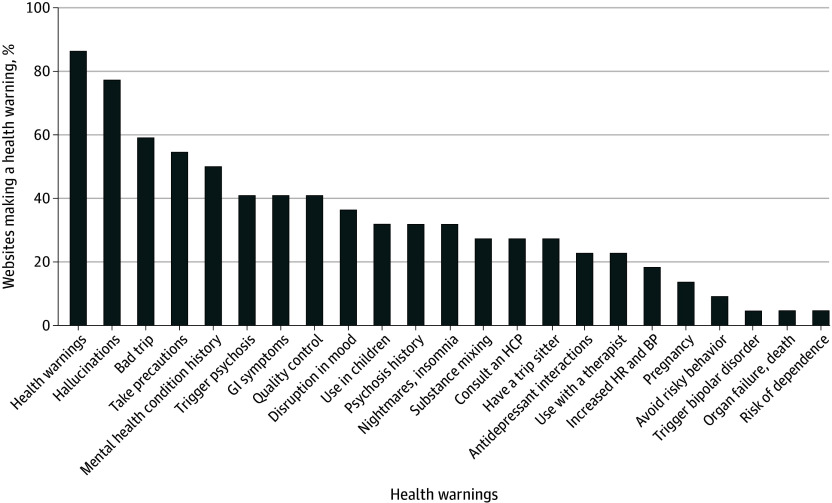
Types of Health Warnings Featured on Psilocybin Websites BP indicates blood pressure; GI, gastrointestinal; HCP, health care professional; HR, heart rate.

Similar to the health claims, 86.4% of the websites made at least 1 health warning (Figure 2). Only 54.5% of the websites warned about psilocybin use in specific risk populations. Of the total number of unique websites (n = 22), 31.8% warned about use in children; 13.6% warned about use in individuals who are pregnant or breastfeeding; 31.8% warned about use in individuals with a history of psychosis, bipolar disorder, or schizophrenia; and 50.0% warned about use in individuals with a history of other mental health conditions.

Most (77.3%) websites cited at least 1 potential risk or adverse effect associated with psilocybin use. The most common adverse effects cited were hallucinations (77.3%), bad trip (eg, increased fear and anxiety) (59.1%), gastrointestinal symptoms (40.9%), and triggering psychosis, latent psychological issues, and paranoia (40.9%). Other potential adverse effects mentioned included disruption in mood (36.4%), nightmares and insomnia (31.8%), and increased heart rate and blood pressure (18.2%).

Most websites also stated safety and use precautions (77.3%). The most prevalent precautions included start of use with a low dose (54.5%); use in the presence of a sober friend (27.3%); consult a health care professional (27.3%); do not mix with alcohol, cannabis, or other substances (27.3%); and do not mix with antidepressants or mood stabilizers (22.7%). In comparison, only 9.1% of websites stated the dangers of driving, operating heavy machinery, or engaging in risky behavior. In addition, 40.9% of websites made product safety claims, including stringent quality control measures, food-grade care, and regular inspections and testing among others.

## Discussion

We identified 57 psilocybin stores operating in 35.7% of major urban centers in Canada as of May 2024, consistent with media reports on proliferation of brick-and-mortar dispensaries.^17,18,19,20,21^ Dispensaries were almost entirely limited to 2 Canadian provinces, Ontario and British Columbia, which had 96.5% of psilocybin stores in Canada but only contain 52.5% of the population of Canada. During the study period, 11 stores that were open in November 2023 had closed by May 2024, most of which (72.7% [n = 8]) were independent stores or part of small chains. Of the 57 stores that remained in operation, most were part of organized and professionalized chains, with a single national chain operating 31.6% (18 of 57) of stores. Most psilocybin retailers had the presence of websites with online delivery, sold a variety of psilocybin formats, and made unverified health claims about the therapeutic benefits of psilocybin use.

Our study documents widespread retail and online access to psilocybin sales along with health benefit claims and/or product promotion. Increasing availability of psilocybin stores matches data indicating rising demand in North America. While recent data in Canada are lacking, between 2013 and 2019, hallucinogen use almost tripled from 0.6% to 2%, and at present, an estimated 6% of Canadians aged 20 to 24 years reported using hallucinogens in the past year.^12^ Research suggests that individuals are using psilocybin for both nonmedical (fun, self-exploration, general mental well-being, and personal growth) and therapeutic purposes, particularly in North America.^35,36^

While psilocybin is illegal in Canada under the Controlled Drugs and Substances Act, the presence of a large psilocybin retail market is indicative of the gray-market status of psilocybin sales nationally.^24,37^ Most Canadian provinces and cities had no identified psilocybin dispensaries, despite presumably relatively uniform demand for psilocybin across the country. This finding mirrors the distribution of illicit cannabis markets in Canada in the period prior to cannabis legalization and may be explained by regional variation in enforcement across the country, with anecdotal reports that many municipal police forces do not consider enforcement a priority.^30^ Evidence suggests that greater cannabis store access is associated with increased use and harms and further research is needed to investigate whether psilocybin retail access may similarly contribute to differences in use.^38^

Dispensaries offered many forms of psilocybin, including dried mushrooms, microdosing capsules, and a wide variety of edible products. Most stores offered psilocybin-infused edibles with packaging and appearance designed to mimic commercial food products (eg, Nutella or popular chocolate bars). There have been large recent increases in accidental cannabis ingestion events, with data suggesting that they are predominantly seen with the ingestion of cannabis edibles.^39^ Ongoing research is warranted to understand whether similar increases in accidental ingestion of psilocybin products will occur. In addition to accidental ingestion, commercialization of products may increase their appeal, leading to higher rates of use.

The promotion of health and wellness claims on psilocybin websites violates the standards set for prescription drugs where advertising of prescription drugs to the general public for the treatment, prevention, or cure of serious disease is prohibited.^40^ Similarly, the current legal framework for nonmedical cannabis promotion requires ensuring promotional material is not seen by minors, prohibits health or cosmetic claims, and mandates the inclusion of health warning labels on cannabis retail products.^41^ Widespread unsubstantiated health claims made on psilocybin dispensary websites have potential negative health implications for the public. First, the lack of regulation or enforcement allows for the spread of possibly false and unproven health claims. For example, one psilocybin dispensary website stated that one of their strains is an excellent medical strain to treat bipolar disorder. While some active clinical trials are investigating the safety of psilocybin in individuals with bipolar disorder, to our knowledge, no study has looked at the efficacy of psilocybin in treating this condition.^42^ Second, dispensaries may be downplaying risks associated with use. For example, less than half of psilocybin websites in this study included a warning about psilocybin use in children, and few warned about use during pregnancy or while breastfeeding. Third, websites frequently misrepresent existing evidence. Most clinical trials conducted to date that have investigated the efficacy of psilocybin in the treatment of depression have involved psychotherapy or psychological support.^43,44,45,46,47,48,49^ However, the psilocybin dispensary websites identified in our study very rarely mentioned that these beneficial effects were most often found in the context of psychedelic-assisted therapy. This discrepancy may lead consumers to believe that there is evidence that psilocybin in isolation has beneficial effects in the treatment of depression. Fourth, while almost half of the dispensaries made product safety claims related to inspection or quality control, no details were provided and the safety and contents of products offered on websites are unverified.

Collectively, our findings highlight the need for policymakers and regulations to catch up with increasing access to the unregulated psilocybin market. Of particular concern is the blurring between medical and nonmedical use of psilocybin, which are distinct types of use that would benefit from separate regulatory approaches and societal discussions.^50^

### Limitations

This study has several limitations. First, our study only collected data cross-sectionally at 2 time points 6 months apart. Psilocybin dispensaries in Canada are dynamic, and between our 2 data collections, 11 dispensaries closed. Consequently, our findings may not characterize future access, and ongoing monitoring is warranted. Second, this study focused on brick-and-mortar psilocybin retailers, representing just one of several access points for Canadians seeking psilocybin, including online delivery services, street dealers, and home cultivation.^35^ While we identified and characterized online sales that were associated with physical retailers, extended research characterizing the total online retail space is warranted. Third, our identification of psilocybin dispensaries depended on their web or media presence. As a result, some brick-and-mortar dispensaries not listed on these platforms may have been overlooked, while others that have since closed may have appeared active based on outdated information from the search engine. Nevertheless, similar methods using map websites have been applied in various studies aiming to map the landscape of vape and cannabis dispensaries.^30,31,32^

## Conclusions

This cross-sectional study found that unlicensed psilocybin dispensaries are currently present in 35.7% of cities in Canada. Most dispensaries are part of organized chains with access to online ordering and delivery and processed psilocybin-infused edible products (eg, candies and chocolates). Dispensaries made a wide variety of health claims about the benefits of psilocybin use, which remain unsupported by current scientific evidence. There is a need to continue monitoring the growth of the emerging unlicensed psilocybin market, alongside related product use and outcomes. In the interim, a variety of measures to address the emerging unregulated psilocybin market should be considered.

## Notes

Supplement 1. **eTable.** Health Statements Featured on Psilocybin Websites

Supplement 2.
Data Sharing Statement

## References

[1] Lieberman JA. Back to the future—the therapeutic potential of psychedelic drugs. N Engl J Med. 2021;384(15):1460-1461. doi:10.1056/NEJMe2102835 33852784

[2] Solmi M, Chen C, Daure C, . A century of research on psychedelics: a scientometric analysis on trends and knowledge maps of hallucinogens, entactogens, entheogens and dissociative drugs. Eur Neuropsychopharmacol. 2022;64:44-60. doi:10.1016/j.euroneuro.2022.09.004 36191546

[3] Nichols DE. Hallucinogens. Pharmacol Ther. 2004;101(2):131-181. doi:10.1016/j.pharmthera.2003.11.002 14761703

[4] Krebs TS, Johansen PØ. Lysergic acid diethylamide (LSD) for alcoholism: meta-analysis of randomized controlled trials. J Psychopharmacol. 2012;26(7):994-1002. doi:10.1177/0269881112439253 22406913

[5] Zeifman RJ, Yu D, Singhal N, Wang G, Nayak SM, Weissman CR. Corrigendum to decreases in suicidality following psychedelic therapy: a meta-analysis of individual patient data across clinical trials. J Clin Psychiatry. 2022;83(3):40898. doi:10.4088/JCP.22l14505 35044730

[6] Smith KW, Sicignano DJ, Hernandez AV, White CM. MDMA-assisted psychotherapy for treatment of posttraumatic stress disorder: a systematic review with meta-analysis. J Clin Pharmacol. 2022;62(4):463-471. doi:10.1002/jcph.1995 34708874

[7] Romeo B, Karila L, Martelli C, Benyamina A. Efficacy of psychedelic treatments on depressive symptoms: a meta-analysis. J Psychopharmacol. 2020;34(10):1079-1085. doi:10.1177/0269881120919957 32448048

[8] Bogenschutz MP, Ross S, Bhatt S, . Percentage of heavy drinking days following psilocybin-assisted psychotherapy vs placebo in the treatment of adult patients with alcohol use disorder: a randomized clinical trial. JAMA Psychiatry. 2022;79(10):953-962. doi:10.1001/jamapsychiatry.2022.2096 36001306 PMC9403854

[9] Goodwin GM, Aaronson ST, Alvarez O, . Single-dose psilocybin for a treatment-resistant episode of major depression. N Engl J Med. 2022;387(18):1637-1648. doi:10.1056/NEJMoa2206443 36322843

[10] Aday JS, Carhart-Harris RL, Woolley JD. Emerging challenges for psychedelic therapy. JAMA Psychiatry. 2023;80(6):533-534. doi:10.1001/jamapsychiatry.2023.0549 37074690

[11] Norring SA, Spigarelli MG. The promise of therapeutic psilocybin: an evaluation of the 134 clinical trials, 54 potential indications, and 0 marketing approvals on ClinicalTrials.gov. Drug Des Dev Ther. 2024;18:1143-1151. doi:10.2147/DDDT.S443177 38618282 PMC11016263

[12] Canada.ca. Canadian Alcohol and Drugs Survey (CADS): 2019 detailed tables. 2019. Accessed June 13, 2024. https://www.canada.ca/en/health-canada/services/canadian-alcohol-drugs-survey/2019-summary.html

[13] Canada.ca. Canadian Alcohol and Drug Use Monitoring Survey. 2012. Accessed June 13, 2024. https://www.canada.ca/en/health-canada/services/canadian-alcohol-drugs-survey/canadian-alcohol-drug-use-monitoring-survey-summary-results-2012.html

[14] Patrick ME, Miech RA, Johnston LD, O’Malley PM. Monitoring the Future Panel Study Annual Report. Institute for Social Research, University of Michigan; 2023. Accessed June 13, 2024. https://monitoringthefuture.org/wp-content/uploads/2023/07/mtfpanel2023.pdf

[15] Livne O, Shmulewitz D, Walsh C, Hasin DS. Adolescent and adult time trends in US hallucinogen use, 2002-19: any use, and use of ecstasy, LSD and PCP. Addiction. 2022;117(12):3099-3109. doi:10.1111/add.15987 35978453 PMC9994631

[16] Keyes KM, Patrick ME. Hallucinogen use among young adults ages 19-30 in the United States: changes from 2018 to 2021. Addiction. 2023;118(12):2449-2454. doi:10.1111/add.16259 37287110

[17] Shetty A. Illegal magic mushroom retail chain expands to Kitchener. CBC News. March 12, 2024. Accessed June 13, 2024. https://www.cbc.ca/news/canada/kitchener-waterloo/magic-mushrooms-funguyz-kitchener-location-1.7139393

[18] Ballard J. Magic mushroom dispensaries operating openly in Vancouver. CBC News. March 16, 2022. Accessed June 13, 2024. https://www.cbc.ca/news/canada/british-columbia/magic-mushroom-dispensaries-in-vancouver-1.6385792

[19] Hasse J. Psychedelic mushroom shops reach the Americas. *Forbes*. June 2, 2021. Accessed June 13, 2024. https://www.forbes.com/sites/javierhasse/2021/06/02/psychedelic-mushroom-shops-reach-the-americas/

[20] Donnini A. Magic mushroom dispensaries multiplying in southwestern Ontario, with no cap in sight. CBC News. August 17, 2003. Accessed June 13, 2024. https://www.cbc.ca/news/canada/london/st-thomas-london-magic-mushroom-dispensary-1.6938408

[21] Stober E. Magic mushrooms are still illegal in Canada. How can stores be opening? Global News. September 4, 2023. Accessed June 11, 2024. https://globalnews.ca/news/9932718/magic-mushrooms-shops-canada/

[22] Wiener S. The Regulated Psychedelic Facilitators Act and the Regulated Psychedelic-Assisted Therapy Act. May 16, 2024. Accessed June 26, 2024. https://legiscan.com/CA/text/SB1012/id/2915482

[23] Drug Fact Sheet. Psilocybin. US Drug Enforcement Administration. April 2020. Accessed June 26, 2024. https://www.dea.gov/sites/default/files/2020-06/Psilocybin-2020.pdf

[24] Psilocybin and psilocin (magic mushrooms). Government of Canada. April 2024. Accessed June 26, 2024. https://www.canada.ca/en/health-canada/services/substance-use/controlled-illegal-drugs/magic-mushrooms.html

[25] Kane L. Regulators urged to include so-called grey market in marijuana legalization. CTV News. February 2018. Accessed June 27, 2024. https://vancouverisland.ctvnews.ca/business/article/regulators-urged-to-include-so-calledgrey-market-in-marijuana-legalization/

[26] Natural Medicine Health Act–DORA implementation timeframe. Accessed June 27, 2024. https://dpo.colorado.gov/NaturalMedicine/Implementation

[27] Oregon Health Authority. Oregon psilocybin services. Accessed June 27, 2024. https://www.oregon.gov/oha/ph/preventionwellness/pages/oregon-psilocybin-services.aspx

[28] Korthuis PT, Hoffman K, Wilson-Poe AR, . Developing the open psychedelic evaluation nexus consensus measures for assessment of supervised psilocybin services: an e-Delphi study. J Psychopharmacol. 2024;38(8):761-768. doi:10.1177/02698811241257839 38888164

[29] Health Canada. Notice to stakeholders: requests to the Special Access Program (SAP) involving psychedelic-assisted psychotherapy. February 27, 2023. Accessed July 9, 2024. https://www.canada.ca/en/health-canada/services/drugs-health-products/drug-products/announcements/requests-special-access-program-psychedelic-assisted-psychotherapy.html

[30] Mahamad S, Hammond D. Retail price and availability of illicit cannabis in Canada. Addict Behav. 2019;90:402-408. doi:10.1016/j.addbeh.2018.12.001 30530299

[31] Lee JG, D’Angelo H, Kuteh JD, Martin RJ. Identification of vape shops in two North Carolina counties: an approach for states without retailer licensing. Int J Environ Res Public Health. 2016;13(11):1050. doi:10.3390/ijerph13111050 27801793 PMC5129260

[32] Pedersen ER, Zander-Cotugno M, Shih RA, Tucker JS, Dunbar MS, D’Amico EJ. Online methods for locating medical marijuana dispensaries: practical considerations for future research. Cannabis. 2018;1(2):22-35. doi:10.26828/cannabis.2018.02.003 31304464 PMC6625809

[33] Statistics Canada. Dissemination area (DA): dictionary, census of population. 2021. Accessed July 10, 2024. https://www12.statcan.gc.ca/census-recensement/2021/ref/dict/az/definition-eng.cfm?ID=geo021

[34] Friesen EL, Konikoff L, Dickson S, Myran DT. Geographic clustering of cannabis stores in Canadian cities: a spatial analysis of the legal cannabis market 4 years post-legalisation. Drug Alcohol Rev. 2024;43(7):1753-1763. doi:10.1111/dar.13869 38803128

[35] Lake S, Lucas P. The Canadian Psychedelic Survey: characteristics, patterns of use, and access in a large sample of people who use psychedelic drugs. Psychedelic Med (New Rochelle). 2023;1(2):98-110. doi:10.1089/psymed.2023.0002 40046727 PMC11658674

[36] Lake S, Lucas P. The Global Psychedelic Survey: consumer characteristics, patterns of use, and access in primarily anglophone regions around the world. Int J Drug Policy. 2024;130:104507. doi:10.1016/j.drugpo.2024.104507 38936219

[37] Donnini A. London’s 1st magic mushroom store, the latest in Ontario, could test limits of authorities’ tolerance. CBC News. May 12, 2023. Accessed October 21, 2024. https://www.cbc.ca/news/canada/london/magic-mushroom-store-funguyz-london-ontario-1.6837278

[38] Cantor N, Silverman M, Gaudreault A, . The association between physical availability of cannabis retail outlets and frequent cannabis use and related health harms: a systematic review. Lancet Reg Health Am. 2024;32:100708. doi:10.1016/j.lana.2024.100708 38486811 PMC10937151

[39] Myran DT, Tanuseputro P, Auger N, Konikoff L, Talarico R, Finkelstein Y. Pediatric hospitalizations for unintentional cannabis poisonings and all-cause poisonings associated with edible cannabis product legalization and sales in Canada. JAMA Health Forum. 2023;4(1):e225041. doi:10.1001/jamahealthforum.2022.5041 36637814 PMC9857209

[40] Health Canada. Illegal marketing of prescription drugs 2020. Accessed October 21, 2024. https://www.canada.ca/en/health-canada/services/drugs-health-products/marketing-drugs-devices/illegal-marketing/prescription-drugs.html#a2

[41] Cannabis health warning messages. Government of Canada. October 17, 2019. Accessed August 26, 2024. https://www.canada.ca/en/health-canada/services/drugs-medication/cannabis/laws-regulations/regulations-support-cannabis-act/health-warning-messages.html

[42] ClinicalTrials.gov. Accessed June 13, 2024. https://clinicaltrials.gov

[43] Watford T, Masood N. Psilocybin, an effective treatment for major depressive disorder in adults—a systematic review. Clin Psychopharmacol Neurosci. 2024;22(1):2-12. doi:10.9758/cpn.23.1120 38247407 PMC10811389

[44] Metaxa AM, Clarke M. Efficacy of psilocybin for treating symptoms of depression: systematic review and meta-analysis. BMJ. 2024;385:e078084. doi:10.1136/bmj-2023-078084 38692686 PMC11062320

[45] Goodwin GM, Malievskaia E, Fonzo GA, Nemeroff CB. Must psilocybin always assist psychotherapy ? Am J Psychiatry. 2024;181(1):20-25. doi:10.1176/appi.ajp.20221043 37434509

[46] Ko K, Kopra EI, Cleare AJ, Rucker JJ. Psychedelic therapy for depressive symptoms: a systematic review and meta-analysis. J Affect Disord. 2023;322:194-204. doi:10.1016/j.jad.2022.09.168 36209780

[47] Davis AK, Barrett FS, May DG, . Effects of psilocybin-assisted therapy on major depressive disorder: a randomized clinical trial. JAMA Psychiatry. 2021;78(5):481-489. doi:10.1001/jamapsychiatry.2020.3285 33146667 PMC7643046

[48] Carhart-Harris R, Giribaldi B, Watts R, . Trial of psilocybin versus escitalopram for depression. N Engl J Med. 2021;384(15):1402-1411. doi:10.1056/NEJMoa2032994 33852780

[49] Gukasyan N, Davis AK, Barrett FS, . Efficacy and safety of psilocybin-assisted treatment for major depressive disorder: prospective 12-month follow-up. J Psychopharmacol. 2022;36(2):151-158. doi:10.1177/02698811211073759 35166158 PMC8864328

[50] Andrews CM, Hall W, Humphreys K, Marsden J. Crafting effective regulatory policies for psychedelics: what can be learned from the case of cannabis? Addiction. 2025;120(2):201-206. doi:10.1111/add.16575 38845381 PMC11707317

