# Supplementary material for: Psilocybin Dispensaries and Online Health Claims in Canada
Source: JAMA Netw Open. 2025 Apr 1;8(4):e252853. doi: 10.1001/jamanetworkopen.2025.2853 (PMC11962669; doi:10.1001/jamanetworkopen.2025.2853)
Supplement: Supplement 1. — eTable. Health Statements Featured on Psilocybin Websites [file jamanetwopen-e252853-s001.pdf]

Supplemental Online Content

Matsukubo J, Dickson S, Xiao J, et al. Psilocybin dispensaries and online health claims in Canada. *J Netw Open*. 2025;8(4):e252853. doi:10.1001/jamanetworkopen.2025.2853

eTable. Health Statements Featured on Psilocybin Websites

This supplemental material has been provided by the authors to give readers additional information about their work.

© 2025 Matsukubo J et al. *JAMA Network Open*.

eTable. Health Statements Featured on Psilocybin Websites

**Health claims or health warnings**	**Quotations**[a]
Medical claims associated with psychedelic-assisted therapy	Research shows promising results in treating mental health conditions. Psilocybin therapy should be conducted with trained professionals and within legal and ethical frameworks. Consult professionals for personalized guidance on magic mushroom use.<br>The [psychedelic integration] therapy offers a different path by using transformative experiences that target the root causes of a person's distress. The evidence points to superior outcomes for specific populations, especially those with conditions like PTSD or severe depression that have not responded well to other treatments.
Anxiety and anxiety disorder	Psilocybin gummies benefits: reduced anxiety (academic, general, or social).<br>The short-term effects of psilocybin on anxiety include a reduction in anxiety symptoms, an increase in positive mood, and a sense of well-being. The long-term effects of psilocybin on anxiety are still being studied, but some studies have shown that psilocybin can have lasting effects on anxiety symptoms.
Depression, MDD, TRD	Studies suggest that Amazonian magic mushrooms can offer relief for those with treatment-resistant depression.<br>Scientists believe that psilocybin, the active compound in magic mushrooms, may reset brain activity patterns associated with depression.
PTSD	Yes, some studies suggest that psilocybin, the active compound in Makilla Gorilla mushrooms, may have potential therapeutic benefits. It has been explored for use in treating issues like depression, anxiety, and PTSD. However, more research is needed for definitive conclusions.<br>Some users utilize blue meanies in therapeutic settings to address issues such as anxiety, depression, and PTSD, under the guidance of a trained professional.
End-of-life distress and anxiety associated with terminal illness	Some individuals have reported that magic mushroom experiences have reduced their fear of death and increased their acceptance of mortality.
SUD (general)	What are the benefits of psilocybin microdoses? ... Weaning off and staying off antidepressants and hard drugs.
AUD	People who have struggled with smoking or alcohol dependence have reported reduced cravings after psilocybin-assisted therapy sessions.
Increased focus and productivity	Our natural, plant-based blend of *Psilocybe cubensis* and adaptogens work in harmony to improve your focus, creativity, and mental clarity, providing a euphoric and energizing experience.
Increased creativity and open-mindedness	Creativity boost: many users experience enhanced creativity and artistic inspiration while under the influence of blue meanies.
Spiritual experience, self-reflection, personal growth, and ego death	Microdosing may promote self-reflection and introspection, which can lead to personal growth and insights into one's behavior and thought patterns.<br>Higher doses will cause intense visuals, spiritual awakening, and ego death.

© 2025 Matsukubo J et al. *JAMA Network Open*.

Wellness flourishing	What are the benefits of psilocybin microdoses? ... improved optimism, mood, spirit, and life appreciation.<br>Some users report improved mood, increased feelings of happiness, and a sense of well-being after using magic mushrooms. These effects can sometimes last for weeks or even months after a single experience.<br>In my own laboratory and through collaborations, I've been part of controlled clinical trials that have delved deep into the therapeutic potential of psilocybin, the primary psychoactive compound in magic mushrooms. The results, while preliminary, have been nothing short of astounding. Under carefully controlled conditions, and with the right therapeutic support, psilocybin has demonstrated a remarkable ability to catalyze profound shifts in mental well-being.
Psychedelic effects	Bull Run offers intense, spiritually enlightening trips with striking visuals. Objects appearing to dance, shift, move, or melt.<br>The effects of Toque are also described as intense and immersive, often marked by vivid visual hallucinations and an altered perception of time and space.
Pain relief	There are anecdotal reports of microdosing helping with chronic pain management, although more research is needed in this area.
Headaches, migraines	Microdosing benefits: reduce physical symptoms (such as muscle tension and headache)
Anti-inflammation and tissue regeneration	These mushrooms have demonstrated potential in enhancing the immune system, reducing inflammation, improving cognitive function, and when micro-dosed, significantly boosting focus.<br>Golden teacher is a very popular ingredient in microdose blends. This strain helps with a wide range of physical, mental, and physiological conditions, such as pain, addiction, anxiety, depression, inflammation, immunity, intimacy, and stress.
Other (eating disorder, insomnia, sleep disorder, OCD, ADHD)	Microdosing can also be used to treat a myriad of mental health issues such as depression, anxiety, ADHD, ADD, PTSD, and others.<br>We have noticed in our research online and from both customer feedback and from employee's own personal medical use that certain types help some mental health diagnoses better than others: Golden Teacher: improved focus and concentration, helps with ADHD & OCD. Blue Meanies: helps reduce anxiety and stress while boosting creativity! Penis Envy: helps those suffering from PTSD, insomnia, and depression.
Caution in children	Store your mushroom products in a safe location and away from children and pets.<br>Keep out of reach of children
Caution in pregnancy/breastfeeding	Do not consume if you are pregnant or breastfeeding<br>The safety of magic mushrooms during pregnancy and breastfeeding is unknown. It's best to err on the side of caution and avoid them.<br>Not recommended for pregnant or nursing folks<br>Magic mushrooms are not for pregnant women, immunocompromised or cardiovascular or neurological patients, and people with mental illnesses. So, people falling into the aforementioned categories should avoid magic mushrooms at all costs to avoid risking their health.

© 2025 Matsukubo J et al. *JAMA Network Open*.

History of psychosis, bipolar disorder, schizophrenia	Those with a history of mental health issues, such as schizophrenia or bipolar disorder, should avoid using magic mushrooms, as they may exacerbate their symptoms.<br>People with a history of mental health issues, particularly conditions like schizophrenia or bipolar disorder, may be more vulnerable to adverse effects. Magic mushrooms can exacerbate these conditions in some cases.
History of mental health conditions	If you are in a deep depressive state or crisis point, do not take a high level dose of mushrooms at that time.<br>Some people with underlying mental health disorders may also wish to avoid microdosing. These people may include those with anxiety disorders, who may find that the practice makes anxiety and traits such as neuroticism worse.
Triggering psychosis, latent schizophrenia, and paranoia	The Jedi Mind F*ck mushrooms do precisely what they are named to do. Isolation or being in uncomfortable situations can worsen its effects; instead of making you euphoric, it will make you paranoid.<br>At very high dosages, risks can also include hallucinations, paranoia, and rarely a temporary drug-induced psychosis.
Bad trip (increased anxiety, panic attacks, fear)	Additionally, some people may experience anxiety or fear during the trip, which can be intense and overwhelming.<br>In rare cases, some individuals may experience a 'bad trip,' which can result in intense feelings of fear, anxiety, and panic. A bad trip can also lead to long-term psychological distress, particularly in individuals with a history of mental health issues.
GI symptoms (nausea, vomiting, cramps)	Some people experience mild nausea<br>You might experience nausea, dizziness, headaches, skin rashes, and other side effects. These symptoms should subside after about 30 min.
Increased HR and BP	Some people may experience physical discomfort after consuming magic mushrooms, including muscle weakness, headache, and increased heart rate.
Nightmares, insomnia	Stop use if you experience irritability or insomnia. Some people may experience drowsiness.
Disruption in mood	Taking too high of a dose of psilocybin can cause powerful feelings. Often positive and healing, but also sometimes feelings of discomfort, increased anxiety, disruption in mood and cognitive impairment.
Hallucinations and altered perception	Magic mushrooms can cause vivid and intense hallucinations, which can be disorienting and confusing.<br>Yes, while many users report positive experiences, potential side effects can include nausea, nervousness, paranoia, and in some cases, hallucinogen-persisting perception disorder (HPPD). It's essential to start with a small dose, especially if you're a beginner.
Potential to cause dependence	Magic mushrooms themselves are not considered addictive, and they do not lead to physical dependence. However, some individuals may develop a psychological dependence on the experience.
Do not mix with alcohol, cannabis, or other substances	Never mix magic mushrooms with other drugs, including alcohol and cannabis. Combining substances can have unpredictable and harmful effects. Do not mix Trinity with alcohol, as it will likely dull the experience.

© 2025 Matsukubo J et al. *JAMA Network Open*.

	Mixing substances can be dangerous and unpredictable. It's generally not recommended, as it can increase the risk of adverse reactions.
Interactions with antidepressants and mood stabilizers	Antidepressants can dramatically reduce the effects of microdosing. Patients taking SSRIs or SNRIs will require microdosing in the range of 200-300 mg for beneficial results. For psychedelic effects, you might need a dose of 2000- 4000 mg.<br>Since psilocybin acts on serotonin receptors, using alongside anything that can impact serotonin function is not recommended (recreational drugs or alcohol). It's especially important not to mix with antidepressants or SSRIs.
Do not drive, swim, climb, or engage in any risky behavior	Do not drive, swim, climb or engage in any risky behavior after taking a high dose.<br>Exercise caution if operating heavy machinery, driving a motor vehicle or involved in activities requiring mental alertness.
Consult a health care professional	It's also important to consult with a health care professional before starting any microdosing regimen.<br>You should also talk to your doctor about the risks and benefits of psilocybin treatment before making a decision.
Start with a low dose	This product comes in 200-mg dose capsules. We recommend first-time users to start with a 200-mg dose (1 capsule). Allow 30-60 min for full effect before another dose is considered.<br>First and foremost, start low and go slow unless you know what dose you can handle.
Have a trip sitter with you	Do not take it on your own. It is best to do this with a guide or at least a companion in a safe space.
Consider using in the presence of a therapist	If you are accessing psilocybin therapy through exemptions or clinical trials, it is recommended to follow the guidance of health care professionals experienced in psychedelic-assisted therapy. They can help ensure safe usage, proper dosing, and provide support throughout the experience.
Quality control assurance	Our products are all handled with extreme, food-grade care. Our facilities are immaculate and our products are regularly inspected and tested by professionals.<br>Our capsules are carefully manufactured in our state-of-the-art laboratory, utilizing cutting-edge technology, and adhering to stringent quality control measures. We prioritize your safety and satisfaction, which is why our capsules undergo rigorous testing to ensure purity, potency, and consistency.

Abbreviations: ADHD, attention-deficit/hyperactivity disorder; AUD, alcohol use disorder; BP, blood pressure; GI, gastrointestinal; HR, heart rate; MDD, major depressive disorder; OCD, obsessive-compulsive disorder; PTSD, posttraumatic stress disorder; SNRIs, serotonin-norepinephrine reuptake inhibitors; SSRIs, selective serotonin reuptake inhibitors; SUD, substance use disorder; TRD, treatment-resistant depression.

[a]The statements are presented verbatim from the psilocybin store websites.

© 2025 Matsukubo J et al. *JAMA Network Open*.
