# Supplementary material for: Psilocybin Dispensaries and Online Health Claims in Canada
Source: JAMA Netw Open. 2025 Apr 1;8(4):e252853. doi: 10.1001/jamanetworkopen.2025.2853 (PMC11962669; doi:10.1001/jamanetworkopen.2025.2853)
Supplement: Supplement 2. — Data Sharing Statement [file jamanetwopen-e252853-s002.pdf]

Data Sharing Statement

Matsukubo. Psilocybin Dispensaries and Online Health Claims in Canada. *JAMA Netw Open*. Published April 01, 2025. doi:10.1001/jamanetworkopen.2025.2853

Data
Data available: No
